# Perlite incorporation for sedimentation reduction and improved properties of high-density geopolymer cement for oil well cementing

**DOI:** 10.1038/s41598-024-60587-5

**Published:** 2024-04-27

**Authors:** Ahmed Abdelaal, Salaheldin Elkatatny, Ahmed Mohsen Abdel Fattah

**Affiliations:** 1 College of Petroleum Engineering and Geosciences, King Fahd University of Petroleum & Minerals, Dhahran, Saudi Arabia; 2 Department of Architecture, King Fahd University of Petroleum & Minerals, Dhahran, Saudi Arabia

**Keywords:** Geopolymers, Perlite, Hematite, Sedimentation, Weighting materials, High-density cement, Engineering, Chemistry, Energy

## Abstract

Portland cement (PC) is known for its environmental and technical concerns and massive energy consumption during manufacturing. Geopolymer cement is a promising technology to totally replace the use of PC in the oil and gas industry. Although geopolymers are widely used in the construction industry, it is yet to see a full-scale application in the petroleum industry. High-density geopolymer cement development is essential to substitute heavy-weight Portland cement slurries for high pressure well cementing applications. Sedimentation issue is associated with high-density cement slurries which use high specific gravity solids such as weighting materials. This problem causes heterogeneity and density variation along the cemented sections. The main target of this work is to evaluate the use of perlite powder to address the sedimentation issue in the heavy weight geopolymer systems. Hematite-based Class F fly ash (FFA) geopolymer cement slurries with perlite concentrations of 0, 1.5, and 3% by weight of binder (BWOB) were prepared. The sedimentation problem was investigated using three techniques: API method, nuclear magnetic resonance (NMR), and computed tomography (CT) scan. The perlite effects on different geopolymer properties such as unconfined compressive strength (UCS), porosity, elastic and rheological properties were assessed. The results proved that perlite incorporation in high-density hematite-based FFA geopolymer significantly reduced sedimentation issue by increasing yield point and gel strength. NMR and CT scan showed that perlite decreases porosity and density variation across the geopolymer samples. The UCS increased with increasing perlite percentage from 0 to 3%. The measured Young’s moduli (YM) and Poisson’s ratios (PR) showed that the developed perlite based geopolymer systems are considered more flexible than Class G cement systems. It was found that the optimum perlite concentration is 3% BWOB for tackling sedimentation and developing a slurry with acceptable mixability and rheological properties.

## Introduction

Portland cement is used in well cementing for various applications such as casing support, zonal isolation, plug & abandonment, loss of circulation control, sidetracking and remedial activities. However, there are specific concerns associated with the properties of PC both before and after cement setting. These concerns include strength retrogression at high temperatures, cement failure due to low flexibility in terms of Poisson’s ratio and Young’s modulus, gas migration, and susceptibility to corrosive environments. The manufacturing process of PC requires significant quantities of natural resources and energy and emits substantial carbon dioxide (CO_2_) emissions. The production of one ton of clinker, a key component of PC, requires approximately 3.2 GJ of energy and generates around 0.93 ton of CO_2_ emissions^1,2^.

Geopolymers have emerged as a promising alternative to traditional Portland cement in a range of applications, including oil well cementing. The term "geopolymer" was originally introduced and coined by Joseph Davidovits^3^. Geopolymers refer to a type of cement-like binder made by the reaction of aluminosilicate materials with alkali hydroxides and/or soluble silicates, resulting in an inorganic polymeric substance^4,5^. More than 65% of the crust of the earth consists of aluminosilicate materials^5^. Aluminosilicate materials encompass a wide range of substances, including both waste materials such as fly ash, red mud, and furnace slag, as well as certain clays like kaolinite and metakaolin.

In the literature, researchers have extensively explored the utilization of waste materials as sources of aluminosilicate materials for geopolymers production. Fly ash, a byproduct of coal combustion, has been widely investigated due to its high content of amorphous aluminosilicates^6,7^. These waste materials not only offer a sustainable alternative to traditional raw materials but also contribute to waste management and environmental sustainability. Additionally, certain clays, such as kaolinite and metakaolin, have been explored for their aluminosilicate content and suitability for geopolymers production^8^. Further investigation and advancements in this area have the potential to contribute to the development of environmentally friendly and technologically advanced materials.

There is no universal cement formulation that can be used for all purposes. Cement slurries are tailored to meet the specific requirements of the downhole environment, considering factors such as temperature, pressure, and cement job type^9^. Heavy-weight cement slurries can be formed either by reducing water to cement ratio or adding weighting agents. Weighting materials are added to drilling fluids and cement slurries to increase their density, thereby enabling safe and effective drilling operations in high pressure oil and gas wells. Hematite, along with other weighting agents, helps control formation pressures, and enhance the performance and integrity of oil well cement. Weighting materials incorporation in geopolymers is a relatively new area in oil and gas well cement. High density hematite based geopolymers suffer from low thickening times and sedimentation issues^10^. Abdelaal et al.^11^ introduced a hematite based FFA geopolymer system for oil well cement. Although the authors provided a mixture of additives to enhance the pumpability of the proposed geopolymer system, the sedimentation evaluation was not a part of their study. The occurrence of solid particles’ settling introduces variations in the compressive strength and bonding across the cemented section. The sedimentation problem in slurries contributes to the formation of a non-uniform cement column, which adversely affects both the strength and zonal isolation.

Perlite is a natural amorphous glassy volcanic rock^12^, which owns a chemical composition equivalent to rhyolite^13^. Chemically, perlite ore contains silica, alumina, and small amounts of several metal oxides^14^. It possesses a high silica content (around 70 wt%) and equilibrium water (2–5 wt%)^13^. Perlite is neutral with a pH of 7.0–7.5^15^. Chemically, perlite is a durable substance capable of maintaining its stability over numerous years^16^; its stability remains relatively unaffected by acids or microorganisms. Recycling perlite presents no environmental concerns due to its inert nature. This rock is formed through the hydration of obsidian. It can be found in various colors, ranging from transparent light gray to glossy black. When subjected to rapid heating at temperatures between 1400 and 1800℉, perlite exhibits a unique property known as expansion or "popping". This expansion process can increase the volume of perlite by a factor of 4 to 20, resulting in a product called expanded perlite^14^. Expanded perlite possesses several desirable physical properties, including low bulk density^17^, good thermal insulation^18^, high fire resistance^18^, low sound transmission, high surface area^17^, and chemical inertness^15^. Both the original perlite rock and the expanded perlite product are commonly referred to as perlite^14^.

Perlite is considered a versatile and environmentally friendly mineral that is extracted and processed with minimal impact on the environment. Perlite can be used in different industrial applications such as sound insulation (sound-absorbing or blocking perlite-based products), high temperature insulation (chimneys, ovens, and foundry applications), filtration (perlite filter aids for food processing, pharmaceuticals, water treatment and fertilizers), construction (plaster and concrete), and horticultural (soilless growing mixes)^19^. The different phases involved in producing expanded perlite yield various waste by-products that have the potential to be utilized in the construction sector, thereby promoting sustainability^20,21^.

Managing waste expanded perlite is challenging due to its remarkably low bulk density, making handling and utilization problematic and contributing to dust formation. Kotwica et al.^21^ mentioned that waste expanded perlite can be used as a supplementary cementitious material (either as a cement replacement or an additive). The authors reported that perlite possesses pozzolanic activity by which it can react with calcium hydroxide and its incorporation enhanced strength.

In the petroleum industry, some researchers evaluated perlite for potential usage in drilling fluids and oil well cement^22–25^. Adjei et al.^23^ investigated the impact of perlite powder on heavy-weight barite cement. It was observed that increasing the concentration of perlite powder led to a decrease in plastic viscosity and an increase in yield point and gel strength. Perlite powder enhanced the 24 h compressive strength and reduced the settling tendency of barite. Basfar et al.^25^ investigated the usage of perlite as an additive in hematite water-based drilling fluids. The results indicated that the addition of perlite provided multiple benefits, including anti-sagging, improved rheological properties, enhanced filtration properties, and minimal changes in mud properties such as pH and density. These findings have implications for improved drilling operations, including better formation protection and reduced pipe sticking issues. Hence, there is a strong motivation to explore the feasibility of incorporating perlite into high-density geopolymer cement systems as well. The primary aim of this study is to assess the effectiveness of utilizing perlite particles in mitigating sedimentation problems within heavy weight geopolymer systems. The secondary objective is to assess the impact of perlite on the properties of Class F fly ash geopolymer cement.

The research in high density geopolymer production for oil and gas industry is limited. This study introduces a new high density geopolymer cement formulation that incorporates two waste materials, namely fly ash and perlite waste, addressing both technical and sustainability concerns in cementing operations. By leveraging these waste materials, the research not only offers technical improvements, such as addressing sedimentation issues, but also highlights significant economic and environmental impacts. The utilization of fly ash and perlite waste materials represents a sustainable approach, reducing the environmental footprint associated with traditional cement production while also offering potential cost savings. This dual focus on technical advancement and sustainability underscores the novelty and importance of this research, positioning it at the forefront of innovation in the field of geopolymer cement technology.

## Materials and methodology

### Materials

**Figure 1:**
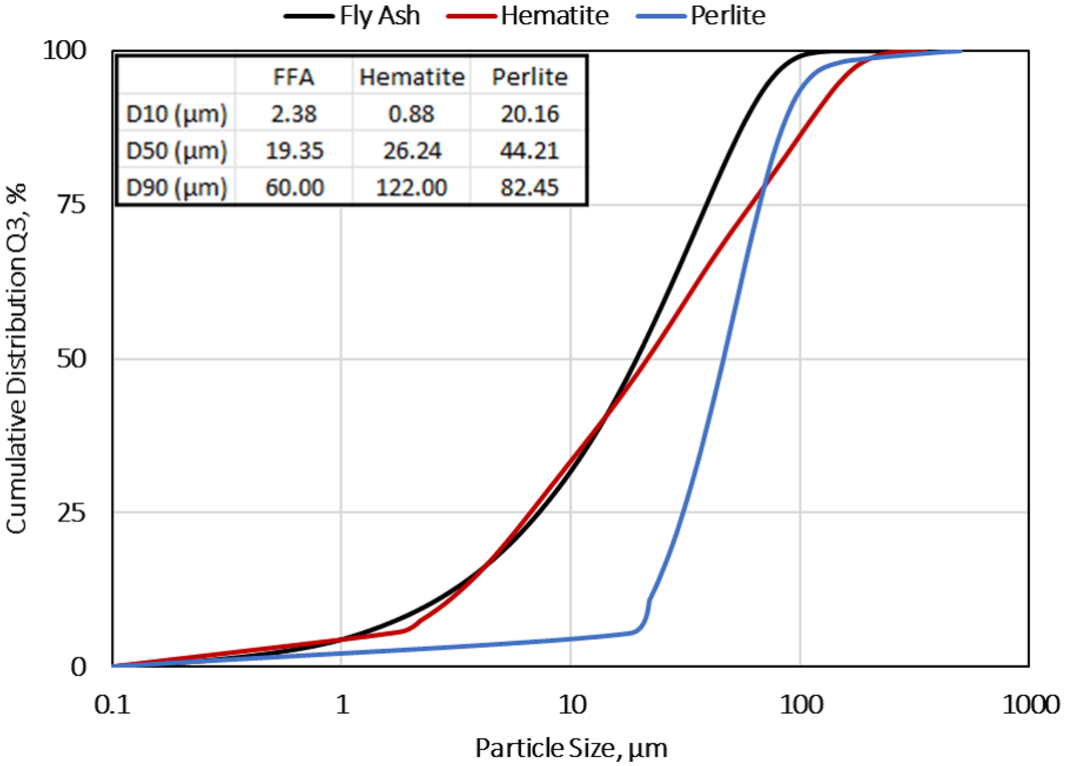
The PSD results of FFA, hematite and perlite.

In this study, various materials were utilized to investigate their suitability for geopolymer applications under wellbore conditions. The aluminosilicate material used was Class F fly ash (FFA), while hematite served as a weighting agent. The activator employed was sodium hydroxide (NaOH) solution, and additional chemical additives such as retarders, deformers, and superplasticizers were included to improve the characteristics of geopolymers. The specific gravities (SG) of FFA and hematite were determined to be 2.25 and 5.05, respectively. Particle size distributions (PSD) were assessed using a laser diffraction particle size analyzer, as depicted in Fig. 1. The findings revealed that 50% of FFA, hematite, and perlite particles had sizes below 19.35 µm, 21.54 µm, and 46.35 µm, respectively. X-ray fluorescence (XRF) analysis confirmed the high iron content (approximately 95%) of hematite, while X-ray diffraction (XRD) analysis confirmed its composition consisting solely of hematite. Furthermore, XRF analysis indicated that FFA contained significant amounts of silica (SiO_2_) and alumina (Al_2_O_3_), as listed in Table 1. These components play a crucial role in the formation of geopolymers. Through comprehensive material characterization, this study provides valuable insights into the composition and properties of FFA, and hematite, and their potential applications in geopolymer formation.

**Table 1:** XRF of FFA used in this work.

Oxide	SiO_2_	Al_2_O_3_	CaO	Fe_2_O_3_	TiO_2_	K_2_O	SO_3_	MnO	Others
FFA	55.92	29.54	5.49	4.93	1.91	1.66	0.39	0.04	0.13

**Figure 2:**
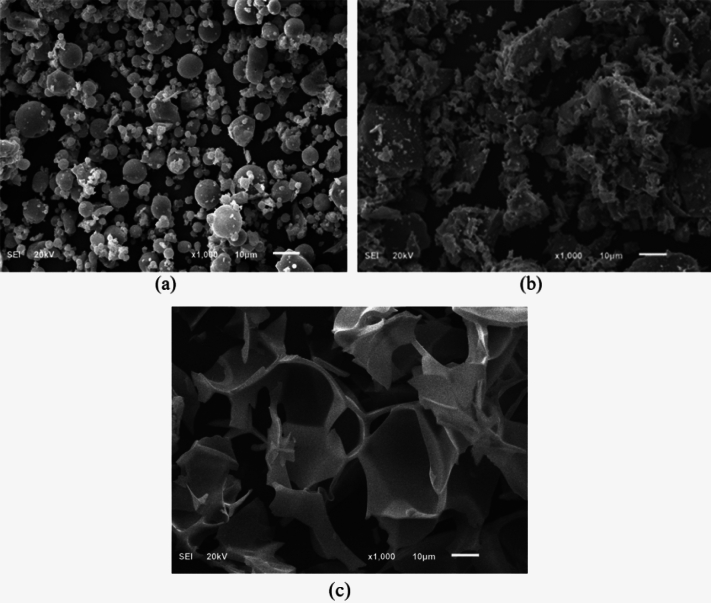
The SEM results for FFA (**a**), hematite (**b**) and perlite (**c**).

The scanning electron microscopy (SEM) images, as shown in Fig. 2, show that the FFA particles possess a spherical shape, the hematite particles have irregular shapes and perlite particles have a flake-like structure. In SEM analysis, perlite is observed to have a distinctive morphology characterized by its porous and lightweight structure. The SEM images reveal an interlocking 3-dimensional structure with numerous interconnected voids and irregular-shaped particles, which contribute to its unique properties. The surface of perlite exhibits a rough texture with a high degree of porosity, allowing for enhanced water absorption and retention capabilities. This unique structure plays a role in minimizing shrinkage during the drying or curing process, thereby preserving the physical dimensions of the material in which they are incorporated. These SEM observations provide valuable insights into the microstructure and surface characteristics of perlite, which are important considerations in its various applications.

### Methodology

**Figure 3:**
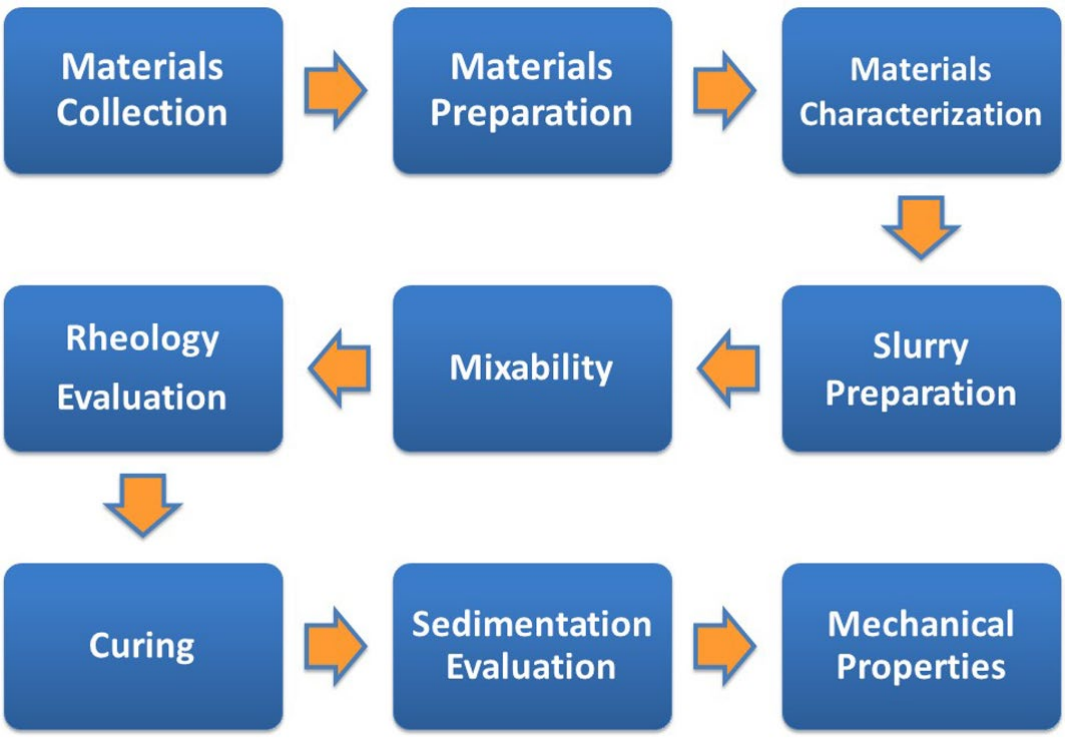
The overview of the steps followed in this work.

This section presents an overview of the methodology employed in this work. The study commenced by gathering, analyzing, and preparing the necessary materials. Subsequently, slurries were prepared and checked for mixability and rheology tests. The sedimentation behavior was assessed through the application of three different techniques. Following this, the cured samples underwent mechanical and elastic properties evaluations. Figure 3 shows an overview of the methods followed in this study.

#### Materials and samples preparation

To prepare the sodium hydroxide (NaOH) solution, NaOH pellets were dissolved in distilled water using a magnetic stirrer to prepare a 4 M NaOH solution. The solution was left to cool at room temperature for a minimum of 24 h before starting the experiment. The specific gravities of the powders were determined using a gas pycnometer. FFA underwent sieving to ensure that the particles’ sizes were below 104 μm, which was confirmed by PSD. In the preparation of geopolymer slurries, two techniques, wet and dry, can be employed^26^. For this study, the wet process technique was utilized. In the wet mixing, superplasticizers and/or retarders are mixed with activation solutions for 2 min at a high shear rate of 12,000 rpm using a mixer. The solid mixture is then added and further mixed with the pre-mixed solution for another 2 min. Three perlite concentrations (0, 1.5 and 3%) were used to prepare the geopolymer slurries as increasing perlite percentage above 3% resulted in a thick slurry with mixability difficulties. The liquid to binder ratio was fixed at 0.56 as used by Abdelaal et al.^11^. The mix design is listed in Table 2. The cured cylindrical samples were 4 inches in length and 1.5 inches in diameter. The samples were cured at 292℉ and 3000 psi for 24 h. The guidelines outlined in the API RP 10B-2^27^ standard were followed in this study, and the modifications made to certain steps have been explicitly addressed to ensure methodological clarity and reproducibility.

**Table 2:** The mix design of the prepared slurries.

Component	BWOB (%)
FFA	100
Hematite	80
Defoamer	0.0164
Superplasticizer	5
Retarder	5
4 M NaOH	56
Perlite	0, 1.5, 3

#### Sedimentation evaluation

Sedimentation evaluation included using three techniques, namely API method, NMR and CT-scan. The cured geopolymer cement samples (4 inches in length and 1.5 inches in diameter) were cut into 3 sections (top, middle and bottom) after curing at 292℉ and 3000 psi for 24 h. The data obtained from the API method, NMR analysis, and CT scan imaging for each section of the geopolymer cement samples were analyzed. Comparative analysis among the sections provided insights into the variation of sedimentation behavior throughout the sample height. The cured geopolymer sections were used to assess sedimentation using the API method. The weight of the geopolymer samples were measured in air and water using a balance. By applying the principle of Archimedes, the specific gravities of the cement sections were calculated by dividing the weights in air by the weights in water. The results are used to construct a density profile for the entire sample^28^.

NMR analysis was conducted to assess sedimentation in the geopolymer cement samples. The cured samples were prepared as cylindrical specimens and placed within the NMR apparatus with a low magnetic field. The NMR measurements were performed to capture the sedimentation behavior through showing the differences in NMR porosity among different cement sections. The resulting NMR data, such as relaxation times, were analyzed to quantify the sedimentation. CT-scan imaging was employed to visualize and analyze the sedimentation behavior of the geopolymer cement samples. The cured samples were scanned using a medical CT scanner, which captured cross-sectional images of the samples. The acquired CT images were processed and analyzed to determine the sedimentation patterns, including the distribution, and settling characteristics of the particles within the samples.

#### Rheological and mechanical properties

After mixing, the geopolymer slurries were conditioned using an atmospheric consistometer at a temperature of 195℉ and a rotational speed of 150 rpm for a duration of 30 min. Rheology evaluation was performed using a viscometer at an average temperature of 195℉ and atmospheric pressure. Rheological properties were evaluated using three different viscometers, and each measurement was repeated three times. The average values obtained from these measurements were then reported. The assessment of mechanical properties included the assessment of unconfined compressive strength (UCS) and dynamic elastic properties such as YM and PR. After conditioning, the slurry was poured into cylindrical molds which were 1.5 inches in diameter and 4 inches in length. Subsequently, the molds were placed in a high temperature and high pressure (HTHP) curing chamber set at 292℉ and 3,000 psi for a duration of 24 h. UCS was estimated using the scratch test method, which involves the continuous shearing action caused by a moving diamond cutter on the surface of the sample. The force exerted on the cutter creates a rock strength profile along the specimen. YM and PR were evaluated by measuring the sonic velocities, specifically the compressional and shear waves. The ultrasonic test measures the time it takes for a pressure wave to travel between two probes.

## Results and discussion

### Sedimentation

**Figure 4:**
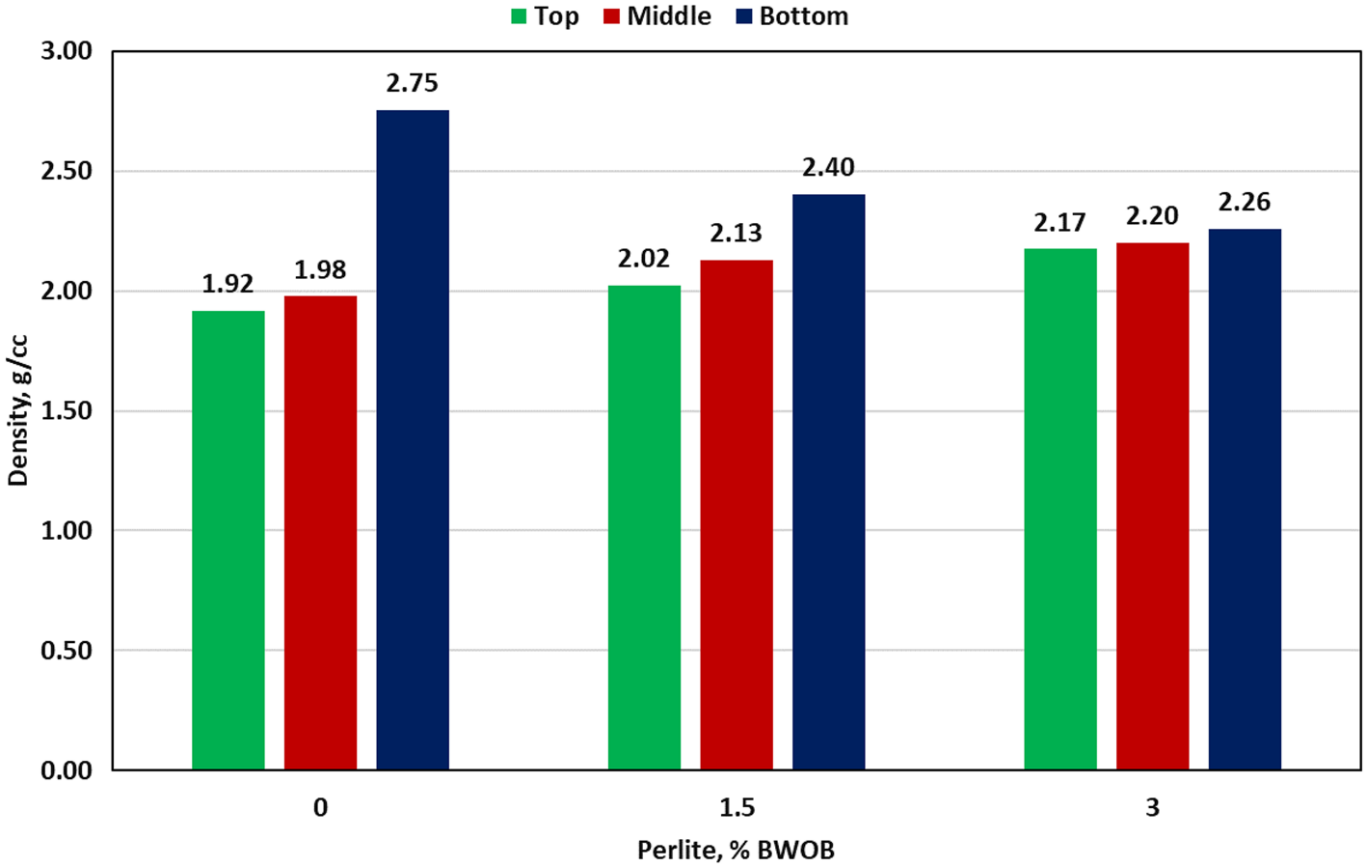
API method results for different perlite concentrations.

**Figure 5:**
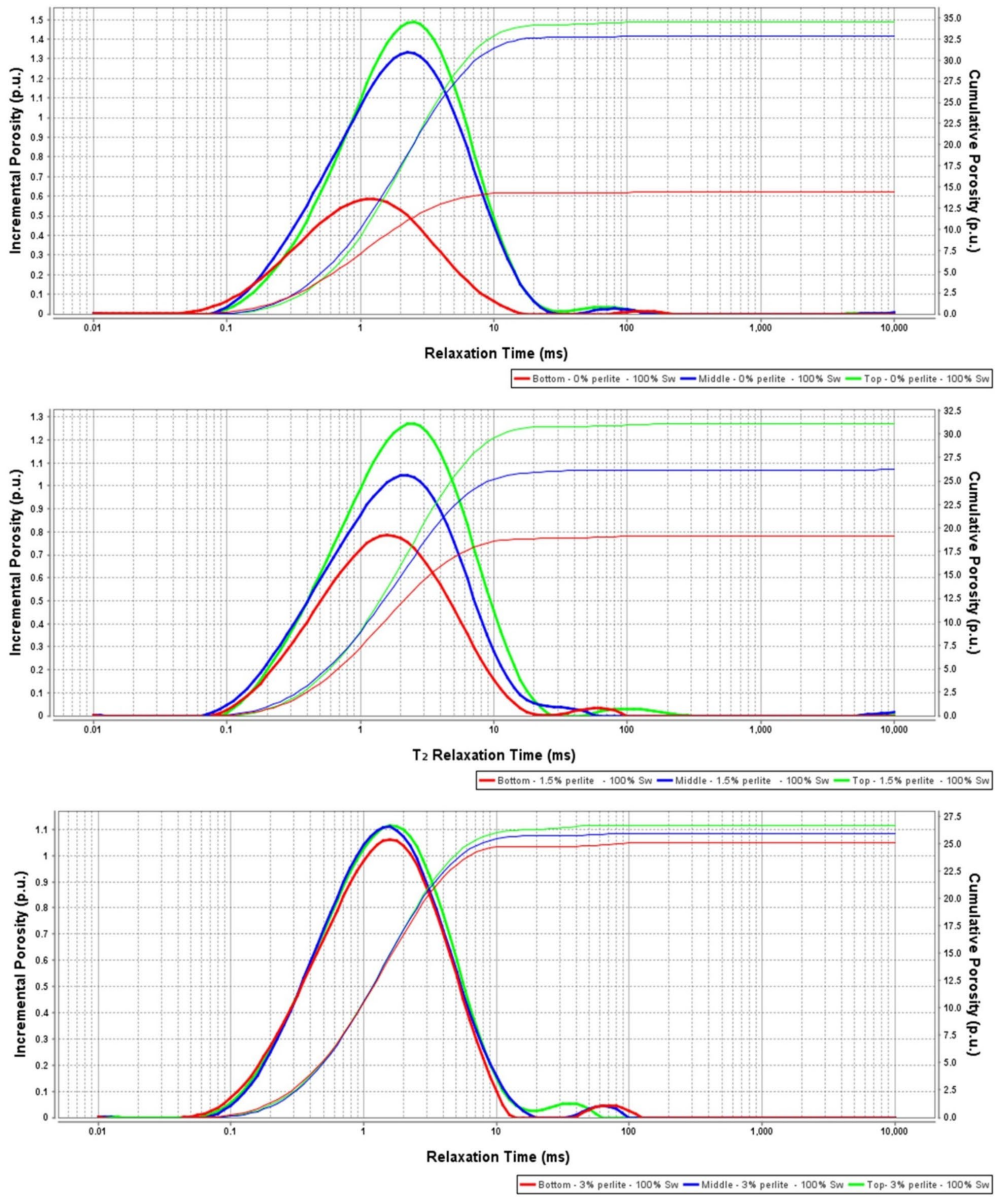
NMR results for the 3 perlite percentages used in this study [0% (**a**), 1.5% (**b**) and 3% (**c**)].

**Figure 6:**
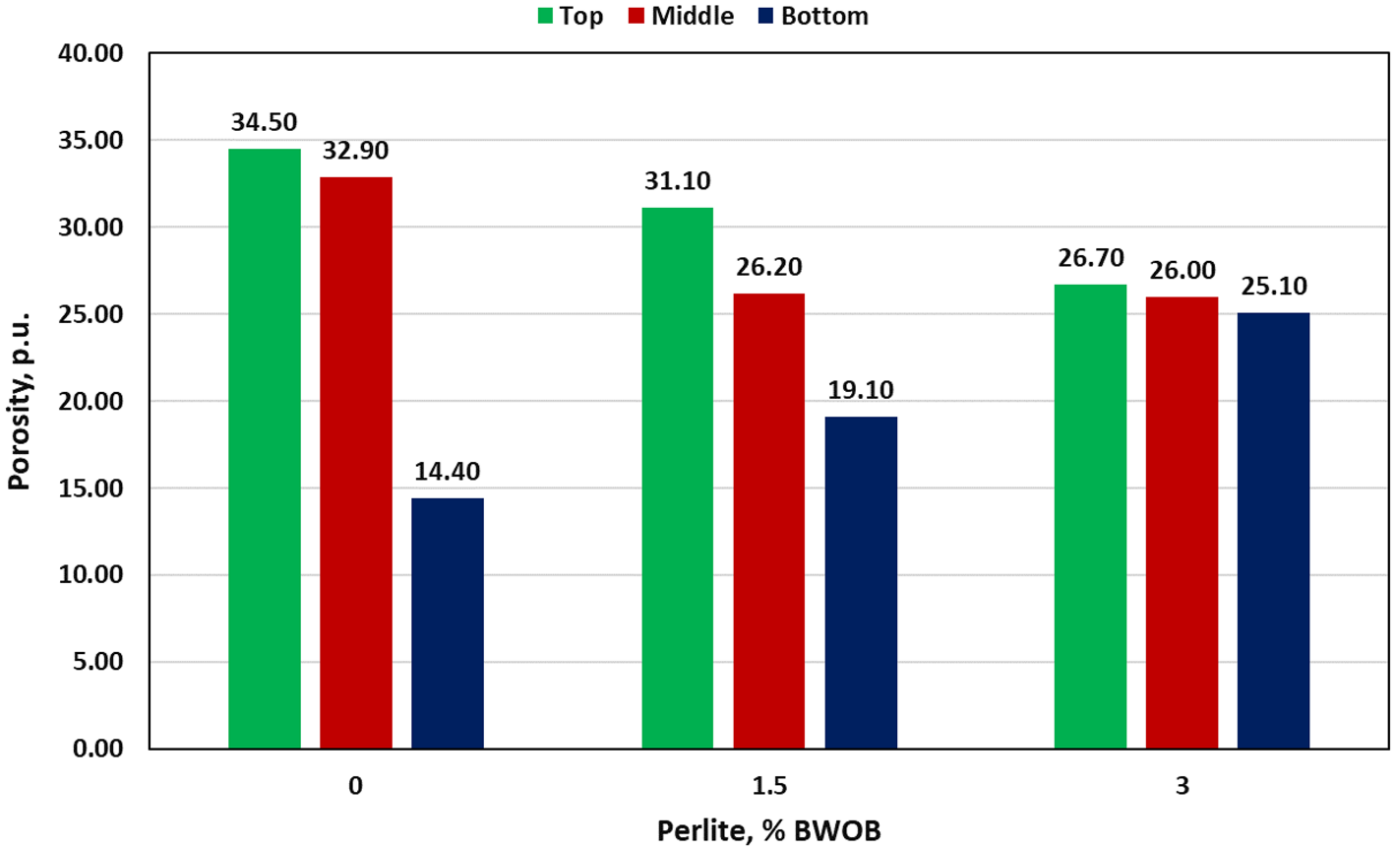
NMR porosities summary for different perlite concentrations.

**Figure 7:**
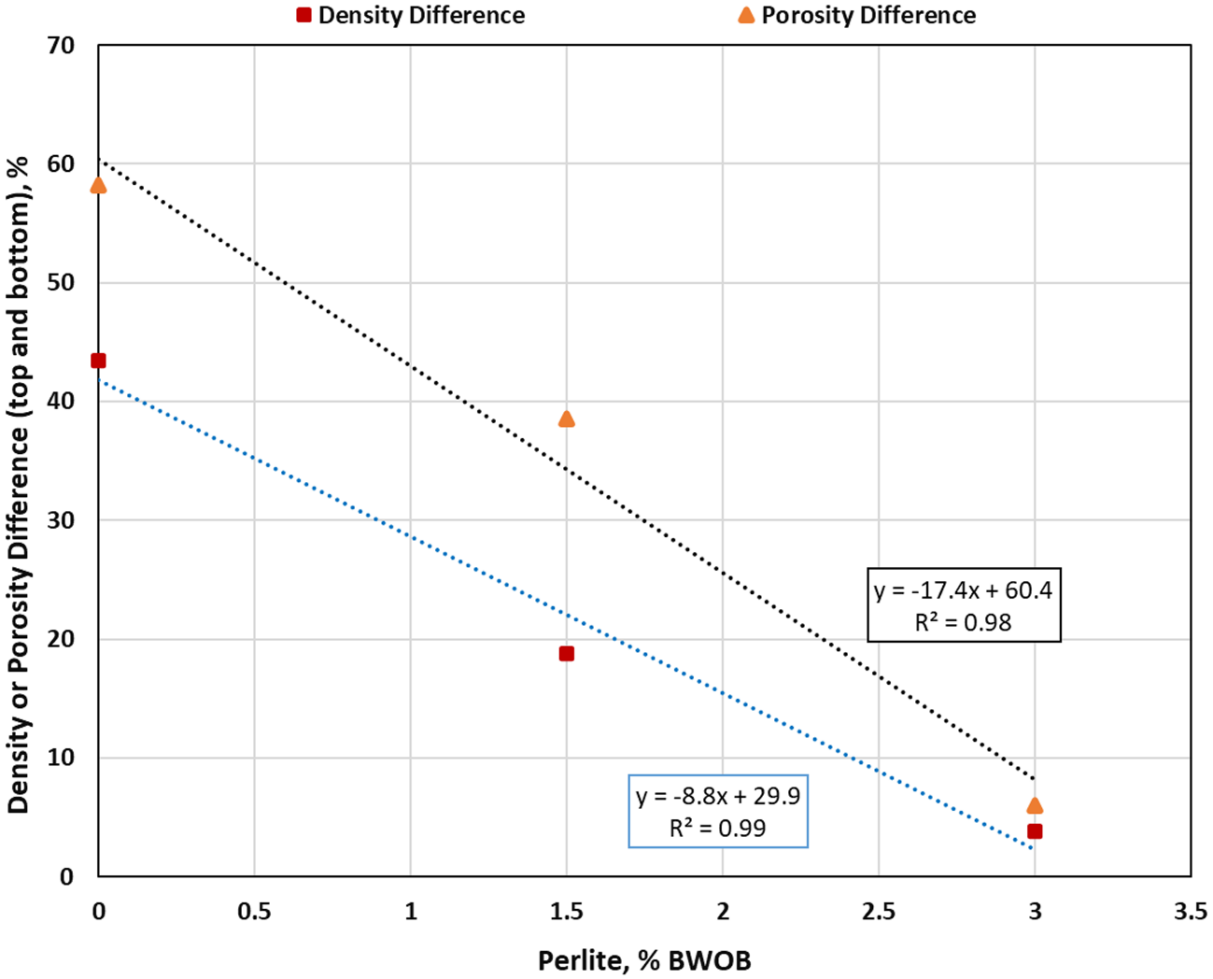
Density and porosity differences between top and bottom sections for different perlite concentrations.

Perlite was introduced to address sedimentation and stability issues accompanying the high-density hematite-based geopolymers. Then, its effects on different cement properties were evaluated. Three perlite percentages (0, 1.5 and 3% BWOB) were used in this study. Adding perlite (3% BWOB) to the system decreased the density variation between the top and bottom sections from 30.3% for 0% perlite to 3.73% for 3% perlite. Figure 4 summarizes the densities of the three sections for each perlite concentration as obtained by the API method. NMR results show that increasing perlite percentage decreased the NMR porosity differences among the three sections of the geopolymer samples (top, middle and bottom) as presented in Fig. 5. Adding perlite (3% BWOB) decreased the top section porosity by 22.6% and increased the bottom section porosity by 74.3% as compared to 0% perlite. The expandable hollow microstructure of perlite particles, as confirmed by SEM, can accommodate FFA and hematite particles within it. Round and spherical particles exhibit faster settling rates in comparison to flat, angular, or irregularly shaped particles. This accelerated settling is a result of reduced friction associated with the smoother, rounded surfaces of the particles^29^. The settling tendency of perlite particles with its content may be deceased due to their irregular shapes. Perlite particles typically have a lower density and larger particle size compared to hematite particles. When perlite is added to the cement system, it increases the overall volume occupied by the particles without significantly increasing the system's weight. This leads to a decrease in the sedimentation rate since the larger and lighter perlite particles tend to remain suspended for longer periods, counteracting the settling tendency of the denser hematite particles. The irregular and porous structure of perlite particles can mechanically interlock with the hematite particles, creating a network that impedes the settling of individual particles. This mechanical entanglement hinders the settling process, leading to reduced sedimentation rates in the cement system. The addition of perlite altered the rheological properties of the slurry, affecting its viscosity and flow behavior. Depending on the concentration and size distribution of the perlite particles, they can modify the viscosity, yield point, and gel strength. These changes in rheology can influence the sedimentation kinetics by affecting the settling velocity of particles and enhancing particle dispersion. The addition of perlite increased both yield point and gel strength of the developed formulations, consequently increasing the suspension capacity. Figures 6 and 7 summarize the NMR porosities, density and porosity differences for different perlite concentrations used in this study.

**Figure 8:**
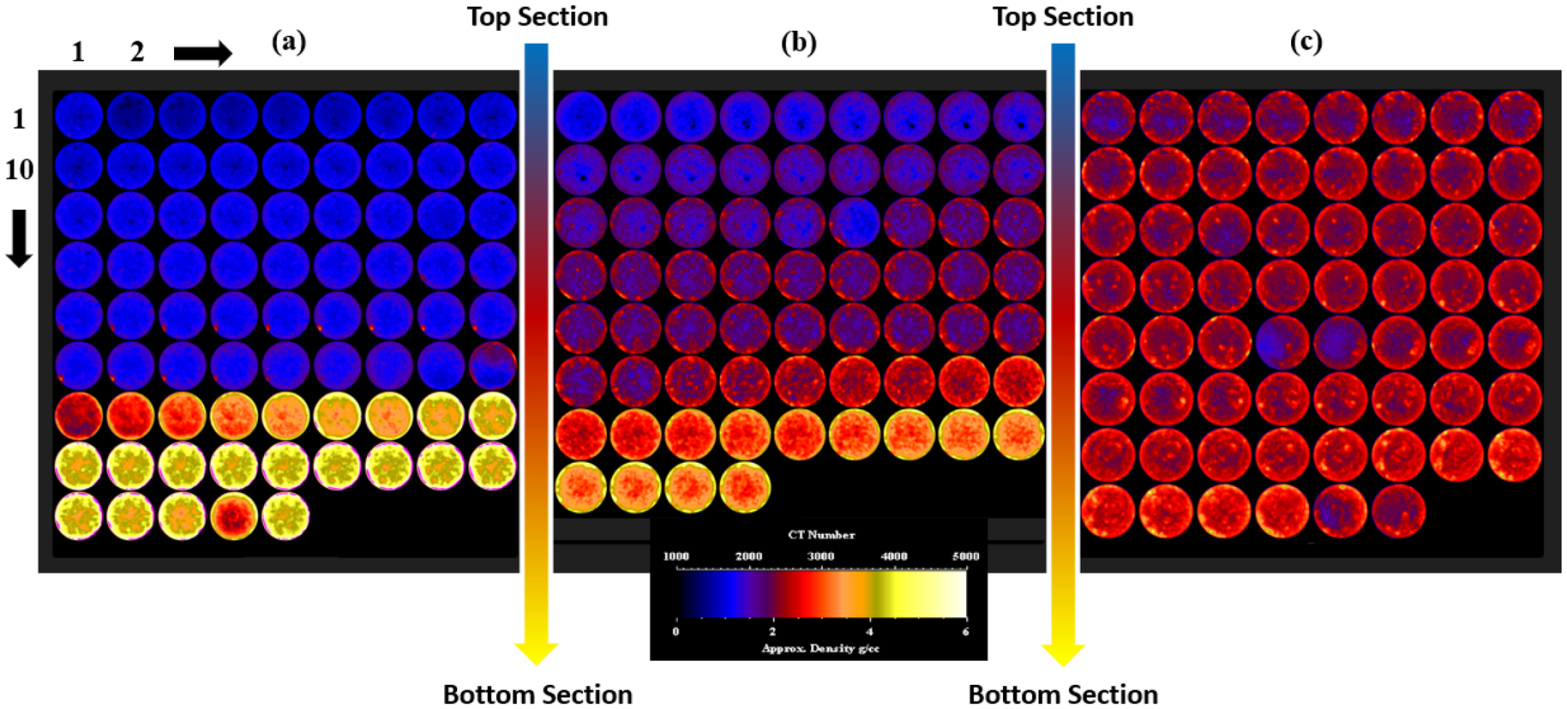
CT-scan results for different perlite concentrations [0% (**a**), 1.5% (**b**) and 3% (**c**)].

The coefficient of determination (R^2^) between perlite concentration and the difference in density or NMR porosity was 0.99 and 0.98 as presented in Fig. 7. There is an agreement between the density and NMR porosity data for different geopolymer cement sections as shown in Figs. 4 and 6. CT-scan imaging confirmed the results obtained by NMR and API method as shown in Fig. 8. There was a remarkable density variation among the slices in Fig. 8a without perlite and this variation decreased by increasing perlite percentage (from left to right in Fig. 8) until getting approximately the same color that indicated minimal density difference across different slices within the same sample as shown in Fig. 8c. The remarkable change in colors from blue (low density) to yellow (high density), as shown in Fig. 8a, may be attributed to the high settling rate and low suspension capacity in the absence of perlite. The color similarity among all slices, as shown in Fig. 8c, indicates homogenous density and porosity along the geopolymer sample from top to bottom after adding 3% BOWB perlite.

### Mechanical properties

**Figure 9:**
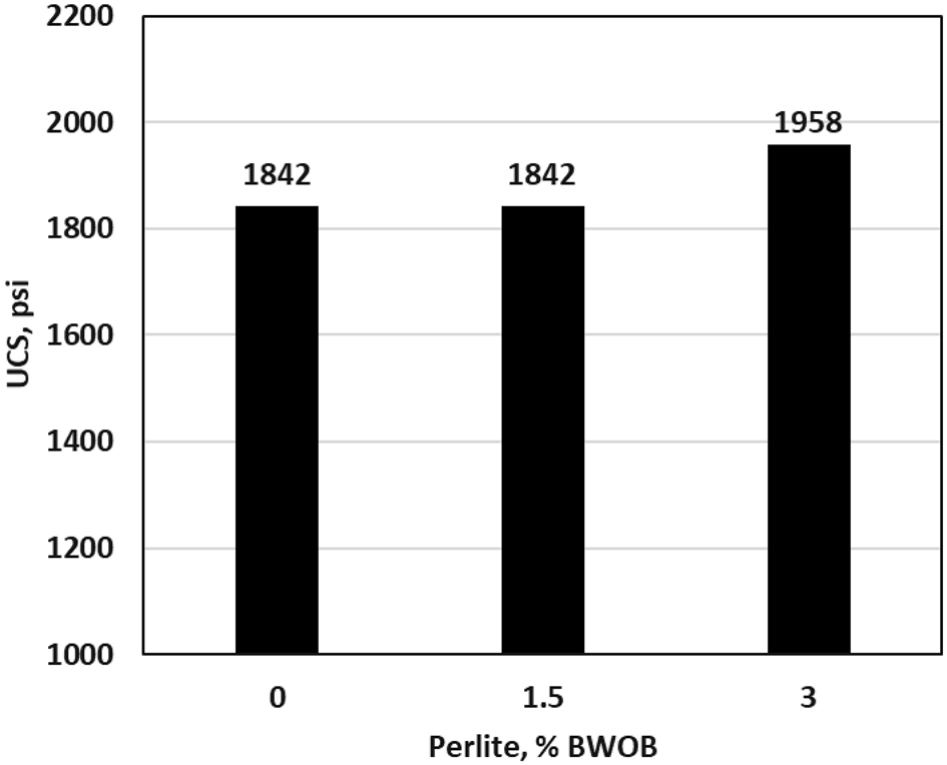
The effect of perlite on UCS.

After ensuring that perlite can be used to address the sedimentation and stability issues, the effects of perlite on other geopolymer properties were assessed. The optimum perlite concentration increased the 24 h UCS of the developed geopolymer as compared to 0% perlite as presented in Fig. 9. The increase in UCS may be attributed to the reduction in the overall porosity of the geopolymer cement samples. Determining the exact compressive strength needed prior to drilling through the casing shoe is challenging. Nonetheless, practical field recommendations suggest a minimum of 500 psi as a suitable guideline^30^. The developed geopolymer formulations showcased a 24 h UCS of at least 1842 psi, exceeding prescribed benchmarks found in literature and some reported values within heavy weight cementing programs.

**Figure 10:**
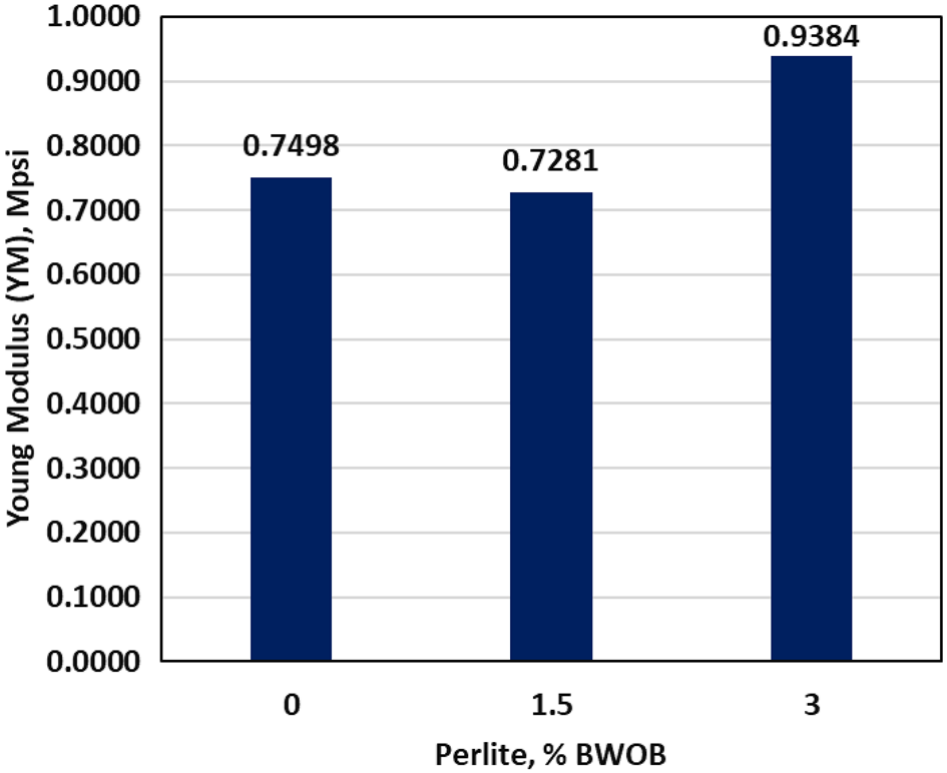
The effect of perlite on YM.

**Figure 11:**
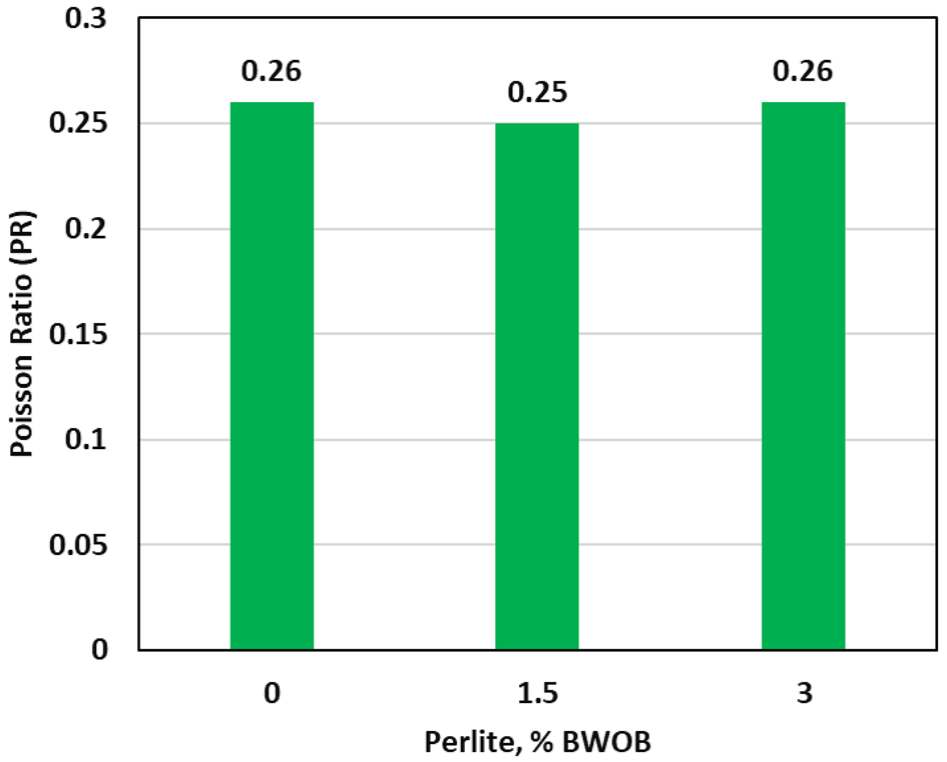
The effect of perlite on PR.

The dynamic YM and PR showed little differences between the geopolymer samples with 0 and 3% perlite concentrations, indicating that the presence of perlite has a minimal effect on the elasticity of the samples as presented by Figs. 10 and 11. A cement with a smaller Young's modulus (YM) is often preferred for unconsolidated strata as it offers better performance compared to a cement with a higher YM^31^. The proposed geopolymer systems possessed a YM in the range of 0.7498 to 0.9384 Mpsi and PR in the range of 0.25 to 0.26 as presented in Figs. 10 and 11. The developed geopolymers exhibit greater flexibility in terms of YM and PR compared to class G cement. As previously mentioned by Liu^31^, the developed geopolymers exhibit lower Young's modulus (YM) compared to shale and consolidated formations. This characteristic makes them suitable for use adjacent to these formations, offering advantages in terms of compatibility and performance. Flexible cement with small YM, large tensile strength, and adequate compressive strength tends to exhibit superior performance in simulating cement stresses. The combination of these properties enables the cement to withstand and adapt to different stress conditions effectively, leading to enhanced durability and overall performance.

### Rheological properties

**Figure 12:**
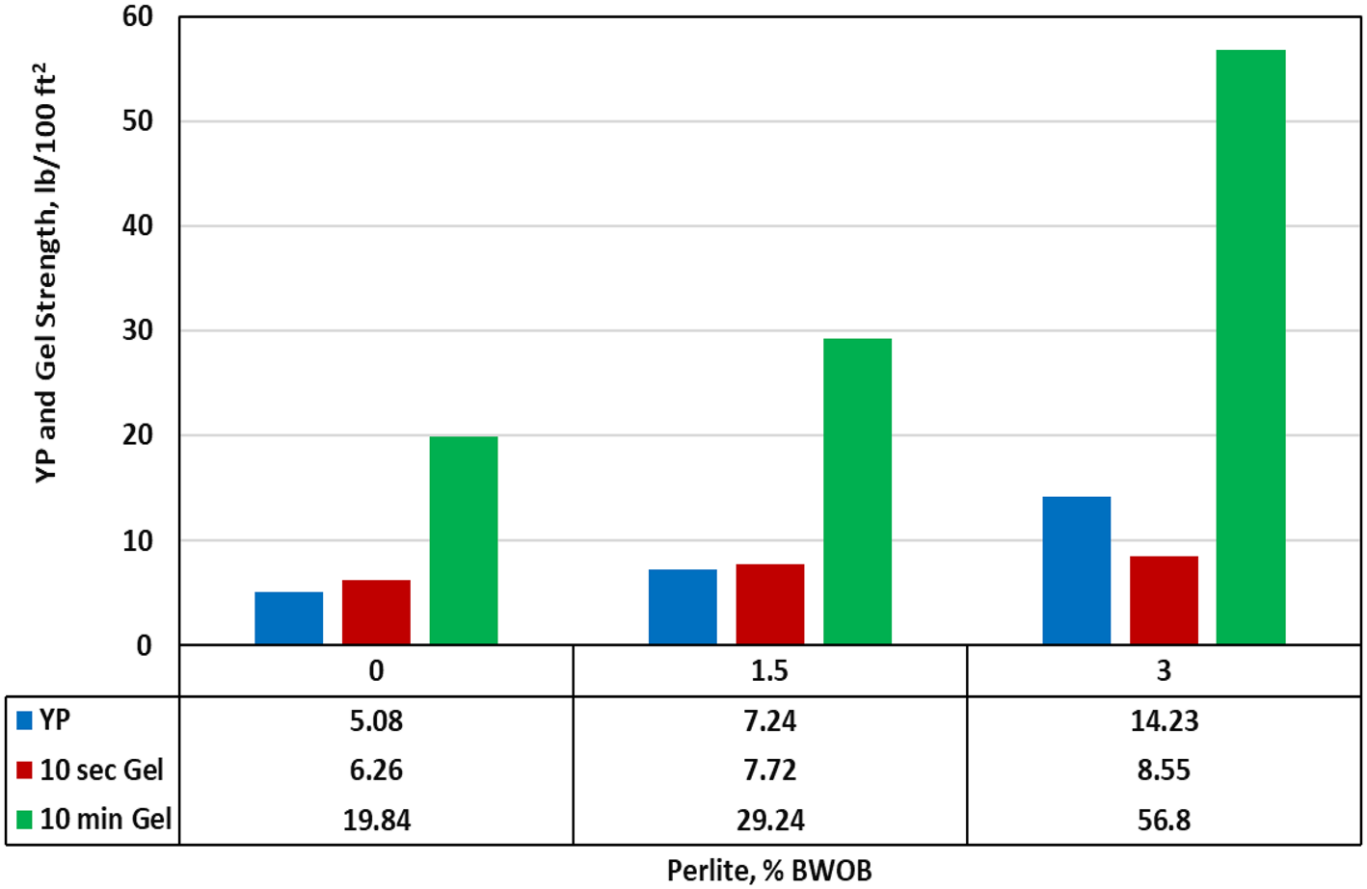
The effect of perlite on rheological parameters.

**Figure 13:**
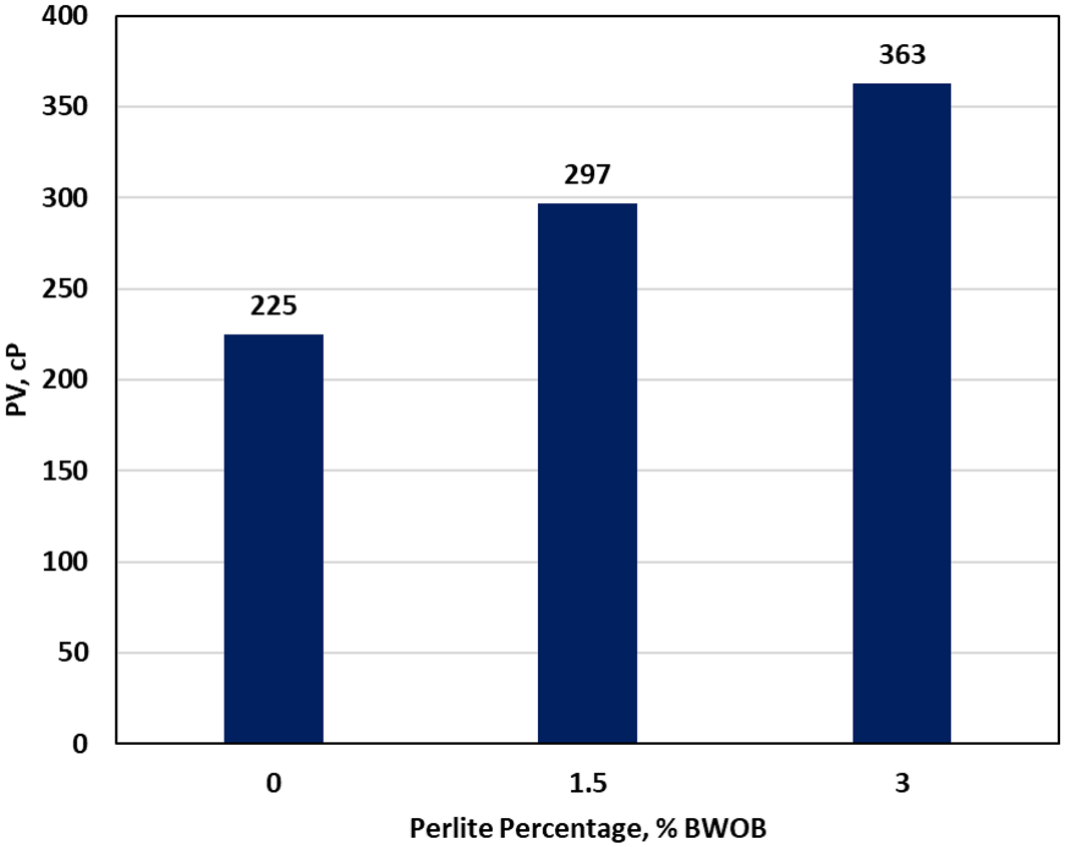
The effect of perlite on plastic viscosity (PV).

The evaluation of rheology in cement slurries is crucial due to its impact on various aspects such as slurry mixing, pumping requirements, and displacement of drilling mud. In this study, the rheology was assessed at an average downhole temperature of 195℉. The rheological analysis of the geopolymer slurries revealed that the Bingham Plastic flow model demonstrated the most accurate fit. This indicates a strong correlation between shear stress and shear rate, confirming the suitability of the Bingham Plastic model for describing the flow characteristics of the slurries. Increasing the perlite percentage increased the yield point by a factor of 1.8 but it adversely affects the plastic viscosity (PV) as it increased PV by 61.3%, as presented in Figs. 12 and 13. The study also examined the gel strength of the developed geopolymer at two-time intervals: 10 s and 10 min. The growth of gel strength plays a significant role in resisting gas invasion. Adding perlite enhanced both 10 s and 10 min gel strength. Figures 12 and 13 provide an overview of the perlite effects on the rheological properties of the developed geopolymer slurries. Both an increase in yield point and gel strength contributed to reducing sedimentation of weighting materials by enhancing the suspension's ability to support and retain the particles in a dispersed state.

### Comparison with existing literature

Partial replacement of hematite with Micromax as a weighting material was introduced as solution for sedimentation issue associated with hematite-based fly ash geopolymer cement systems^32^. This approach was applied under similar downhole conditions and material compositions as those in the current study. In contrast to using perlite waste as an additive up to 3%, Micromax was employed at a 50% partial replacement of hematite. However, it's worth noting that the Micromax solution comes at a higher cost. While the unconfined compressive strength (UCS) was found to be higher in the perlite-based solution, the Micromax solution exhibited a lower plastic viscosity, which is generally considered advantageous. Additionally, the elastic properties between the two solutions were comparable, suggesting similar performance in terms of elasticity and strength. Overall, both solutions offer viable approaches to tackle sedimentation, but the choice between them may depend on specific project requirements, cost considerations, and desired rheological properties.

### Economic and environmental values

The incorporation of fly ash and perlite waste into geopolymer cement systems presents compelling economic advantages. By repurposing these waste materials as valuable inputs, the research contributes to cost savings for cement manufacturers and oil and gas companies. Fly ash, a byproduct of coal combustion, is often available at minimal cost, reducing the overall production expenses associated with cement manufacturing. Similarly, perlite waste, typically generated during processing or manufacturing, represents an opportunity for cost-effective material utilization, further enhancing the economic viability of geopolymer cement production. The utilization of these waste materials not only lowers raw material acquisition costs but also reduces waste disposal expenses, thereby improving the overall economic efficiency of cement production processes.

From an environmental perspective, the utilization of fly ash and perlite waste in geopolymer cement systems offers significant sustainability benefits. By diverting these waste materials from landfills and repurposing them as valuable materials, the research contributes to waste reduction and resource conservation efforts. Moreover, geopolymer cement production generates lower carbon emissions compared to traditional Portland cement manufacturing, aligning with global efforts to mitigate climate change and reduce environmental impact. By promoting the use of fly ash and perlite waste in geopolymer cement systems, the research facilitates the transition towards more sustainable and environmentally friendly cementing practices in the oil and gas industry. Incorporating these economic and environmental perspectives into the paper underscores the added value of the research, demonstrating its broader significance in promoting circular carbon economy, resource efficiency, and environmental responsibility in cement manufacturing and oil well cementing operations.

## Conclusions

In this study, the effectiveness of perlite in addressing sedimentation and stability issues in high-density hematite-based geopolymer systems was investigated. Three different concentrations of perlite (0, 1.5, and 3% BWOB) were evaluated to assess their impact on various cement properties. Overall, the results highlight the significant benefits of incorporating perlite in geopolymer cement formulations, particularly in mitigating sedimentation and stability issues, improving compressive strength, and maintaining desirable rheological properties. These findings contribute to the advancement of geopolymer technology in the oil and gas industry, offering potential solutions for cementing challenges in various wellbore conditions. The study outcomes can be summarized as follows.

- The API method, NMR and CT-scan results demonstrated that the addition of 3% perlite significantly reduced the sedimentation (density and porosity variations) between the top and bottom sections of the geopolymer samples.
- The addition of perlite had a positive effect on the compressive strength of the developed geopolymer, with the optimum perlite concentration leading to a 24-h UCS of at least 1958 psi, surpassing the minimum requirements for drilling applications.
- The Young's modulus and Poisson's ratio showed minimal differences between the geopolymer samples with 0% and 3% perlite concentrations, indicating that the presence of perlite had a negligible effect on the elasticity of the samples.
- Increasing the perlite percentage resulted in higher yield points but adversely affected the plastic viscosity.
- The gel strength of the geopolymer slurries was enhanced by the addition of perlite, both at 10 s and 10 min.

In conclusion, the incorporation of fly ash and perlite waste in geopolymer cement systems not only resolves some technical challenges but also provides economic savings and environmental benefits. This approach promotes sustainable practices in cement manufacturing and contributes to waste reduction efforts in the oil and gas sector.

## Data Availability

No external data was used for this research. All the generated experimental data are included in this manuscript. The datasets used and/or analysed during the current study available from the corresponding author on reasonable request.
